# Forty years of integration of health and social services in the province of Québec (Canada)

**Published:** 2012-09-04

**Authors:** Réjean Hébert

**Affiliations:** Research Center on Aging Sherbrooke University, Québec, Canada

**Keywords:** PRISMA, integrated health and social services, Canada, public insurance, long-term care, structural integration

## Abstract

Québec is the only province in Canada to have integrated health and social services since 1971. A single ministry is responsible for health and social services and this integration is also effective at regional and local agencies. The Local Community Services Centres (*Centre locaux de services communautaires*—CLSC) were created to provide preventive and primary care and services for a borough in large cities, a medium-size town or many villages in a rural area. In 2003, a major reform created the Health and Social Service Centres (*Centre de santé et de services sociaux*—CSSS) by merging hospitals, nursing homes and CLSC in 95 areas over the province.

This structural integration has taken place at the same time that the PRISMA model of coordination-type integration for frail older people was being implemented. Integration improves efficiency of the system, but underfunding of long-term care still hampers the provision of adequate home care services. It is now time for moving the beveridge-type funding system to a long-term care public insurance covering the needs of community-dwelling older people with disabilities.

Powerpoint presentation available at http://www.integratedcare.org at congresses – San Marino – programme.

